# Serological reactivity to *Anaplasma phagocytophilum* in neoehrlichiosis patients

**DOI:** 10.1007/s10096-018-3298-3

**Published:** 2018-06-14

**Authors:** Linda Wass, Anna Grankvist, Mattias Mattsson, Helena Gustafsson, Karen Krogfelt, Björn Olsen, Kenneth Nilsson, Andreas Mårtensson, Hanne Quarsten, Anna J. Henningsson, Christine Wennerås

**Affiliations:** 1 Department of Infectious Diseases, Sahlgrenska Academy, University of Gothenburg, Göteborg, Sweden; 2 Department of Hematology, Uppsala University Hospital, Uppsala, Sweden; 3 Department of Hematology, Gävle Hospital, Gävle, Sweden; 4 Department of Bacteria, Parasites and Fungi, Statens Serum Institut, Copenhagen, Denmark; 5 Section of Clinical Microbiology and Infectious Diseases, Department of Medical Sciences, Uppsala University, Uppsala, Sweden; 6 Department of Women’s and Children’s Health, International Maternal and Child Health, Uppsala University, Uppsala, Sweden; 7 Department of Medical Microbiology, Sørlandet Hospital Health Enterprise, Kristiansand, Norway; 8 Department of Clinical Microbiology, County Hospital Ryhov, Jönköping, Sweden; 9 Department of Clinical Microbiology, Sahlgrenska University Hospital, Guldhedsgatan 10, 413 46 Göteborg, Sweden

## Abstract

The tick-borne bacterium *Candidatus* (Ca.) Neoehrlichia (N.) mikurensis is a cause of “fever of unknown origin” because this strict intracellular pathogen escapes detection by routine blood cultures. Case reports suggest that neoehrlichiosis patients may display serological reactivity to *Anaplasma* (*A*.) *phagocytophilum.* Since *Anaplasma* serology is part of the diagnostic work-up of undetermined fever in European tick-exposed patients, we wanted to investigate (1) the prevalence of *A. phagocytophilum* seropositivity among neoehrlichiosis patients, (2) the frequency of misdiagnosed neoehrlichiosis patients among *A. phagocytophilum* seropositive patients, and (3) the frequency of *A. phagocytophilum* and *Ca.* N. mikurensis co-infections. Neoehrlichiosis patients (*n* = 18) were analyzed for *A. phagocytophilum* IgM and IgG serum antibodies by indirect immunofluorescence assay. Serum samples from suspected anaplasmosis patients (*n* = 101) were analyzed for bacterial DNA contents by singleplex PCR specific for *A. phagocytophilum* and *Ca.* N. mikurensis, respectively. One fifth of the neoehrlichiosis patients (4/18) were seropositive for IgM and/or IgG to *A. phagocytophilum* at the time of diagnosis. Among the patients with suspected anaplasmosis, 2% (2/101) were positive for *Ca.* N. mikurensis by PCR whereas none (0/101) had detectable *A. phagocytophilum* DNA in the serum. To conclude, patients with suspected anaplasmosis may in fact have neoehrlichiosis. We found no evidence of *A. phagocytophilum* and *Ca.* N. mikurensis co-infections in humans with suspected anaplasmosis or confirmed neoehrlichiosis.

## Introduction

*Candidatus* (Ca.) Neoehrlichia (N.) mikurensis is a tick-borne pathogen found in Europe and Asia [1], which was first reported to be a human pathogen in 2010 [2–4]. It can give rise to a severe infectious disease named neoehrlichiosis that features fever and vascular events in immunocompromised patients [5]. Immunocompetent individuals infected by *Ca.* N. mikurensis may present with fever and symptoms indicative of systemic infection, isolated erythematous skin rashes, or no symptoms at all [2, 4, 6–9].

Like all members of the *Anaplasmataceae* family, *Ca.* N. mikurensis is a strict intracellular pathogen, and consequently does not grow in cell-free media, which is why it escapes detection by routine blood cultures [1]. At present, the only microbiological diagnostic option is PCR since there are no serological assays available. The restricted diagnostic alternatives, together with the novelty of this emerging pathogen, explain why many patients with severe neoehrlichiosis remain undiagnosed and fall under the epithet of “fever of unknown origin” [5]. In central and northern Europe, *A. phagocytophilum* serology is part of the diagnostic work-up of (tick-exposed) patients with unexplained fever. There are at least three case reports of immunocompetent patients infected by *Ca.* N. mikurensis who were seropositive for *A. phagocytophilum* as determined by indirect immunofluorescence assay (IFA): two of the cases had de novo production of *Anaplasma*-reactive antibodies, whereas the third one had pre-existing antibodies [2, 6]. This might indicate that *Ca.* N. mikurensis infection can trigger the production of *Anaplasma* cross-reactive antibodies or the occurrence of double infections with *Ca.* N. mikurensis and *A. phagocytophilum.* Consequently, neoehrlichiosis patients may be wrongly diagnosed with anaplasmosis. The aims of this study were to address these issues. Specifically, the goals were to investigate (1) the prevalence of *Anaplasma* seropositivity among patients diagnosed with neoehrlichiosis, (2) whether neoehrlichiosis patients are misdiagnosed as anaplasmosis patients based on serological findings, and (3) the existence of *A. phagocytophilum* and *Ca.* N. mikurensis co-infections.

## Methods

### Study subjects

Eighteen patients diagnosed with neoehrlichiosis based on PCR-positive blood samples were investigated for *A. phagocytophilum*-reactive antibodies in serum (Table 1). Clinical data pertaining to some of these patients have been published previously [3, 5, 10–12]. Serum samples derived from a total of 101 anonymous patients queried for *A. phagocytophilum* antibodies were analyzed by PCR for the presence of bacterial DNA corresponding to *Ca.* N. mikurensis and *A. phagocytophilum*, respectively. The sera were obtained from three clinical microbiology laboratories, two in Sweden (Sahlgrenska University Hospital in Göteborg, *n* = 68, and Ryhov County Hospital in Jönköping, *n* = 22), and one in Denmark (Statens Serum Institut in Copenhagen, *n* = 11). These laboratories are the only ones that perform serological analyses of human antibodies to *A. phagocytophilum* in Sweden and Denmark, respectively. Over half of the blood samples submitted to Sahlgrenska University Hospital (38/68) for analysis of *A. phagocytophilum* antibodies and *Ca.* N. mikurensis DNA were from a prospective study on human tick-borne infections conducted at the Center of Vector-borne Infections, Uppsala University Hospital, Sweden. The study was approved by the local Ethical Review Boards of Göteborg and Uppsala, Sweden. All analyses were performed on thawed blood samples that had been stored frozen at either − 20 °C (sera tested for *A. phagocytophilum* antibodies) or − 120 °C (plasma from neoehrlichiosis patients).

**Table 1:** Clinical characteristics of neoehrlichiosis patients

Patient ID	Age	Sex	Disease	Immune suppression	Fever	Ref.
SE01	77	M	B-chronic lymphocytic leukemia	Splen	Yes	[3]
SE02	75	M	B-chronic lymphocytic leukemia	Splen, Rtx, CH, Co	Yes	[5]
SE03	67	F	Systemic lupus erythematosus	Splen, Co	Yes	[5]
SE05	54	M	Psoriasis, hereditary gout	CH, Co	Yes	[5]
SE06	59	M	Diffuse large B cell lymphoma	Splen, Rtx, CH	Yes	[5]
SE09	78	M	Rheumatoid arthritis	Rtx, CH	Yes	[10]
SE10	55	M	Granulomatosis with polyangiitis	Rtx CH, Co	Yes	[10]
SE12	57	M	Pre-B-acute lymphocytic leukemia	CH, Co	Yes	[10]
SE13	65	F	Autoimmune hemolytic anemia, Crohn’s disease	Splen, Co, Azt	Yes	This study
SE15^a^	57	F	Multiple sclerosis	Rtx	Yes	[11]
SE16	23	M	Healthy	–	No	This study
SE17	58	M	Follicular lymphoma	Rtx, CH	Yes	This study
SE18^a^	69	M	B-chronic lymphocytic leukemia	Splen, Rtx, Co	Yes	This study
SE19	81	M	Polymyalgia rheumatica	Co	Yes	This study
SE20	68	F	B-chronic lymphocytic leukemia	Ibr, CH	Yes	This study
SE21	63	F	Primary hypogammaglobulinemia	Splen	Yes	This study
SE22	60	F	Focal segmental glomerulosclerosis	Co, CyA	Yes	This study
NO01	63	M	Cured Hodgkin lymphoma	Splen	Yes	[12]

*SE* Sweden, *NO* Norway, *M* male, *F* female, *Splen* splenectomy, *Rtx* rituximab, *CH* chemotherapy, *Co* systemic corticosteroids, *Azt* azathioprine, *Ibr* ibrutinib

^a^Diagnosed in retrospect based on clinical data

### *A. phagocytophilum* serology

A commercial IFA assay for analysis of IgG and IgM antibodies to a human isolate of *A. phagocytophilum* (Focus Diagnostics, Cypress, CA, USA) was used according to the manufacturer’s recommendations. Semi-quantitative endpoint antibody titers were obtained by serial twofold dilutions of reactive serum or EDTA-plasma samples. IgM titers ≥ 1:20 were regarded as positive. IgG titers of ≥ 1:64 were regarded as positive at Sahlgrenska University Hospital and Statens Serum Institut in Copenhagen, whereas a cutoff of ≥ 1:80 was employed at Ryhov Hospital in Jönköping using the same assay.

### *Ca.* N. mikurensis PCR

Bacterial DNA was robot extracted (MagNA Pure Compact Extraction Robot, Roche, Basel, Switzerland) from 400 μL of serum or EDTA-plasma (Nucleic Acid Isolation Kit I, Roche) and analyzed by using a real-time TaqMan PCR specific for a 169-bp segment of the *groEL* gene of *Ca.* N. mikurensis as previously described [6]. A synthetic plasmid containing the 169-bp sequence cloned into a pUC57 vector (Genscript, Piscataway, NJ, USA) was used to establish a standard curve and the limit of detection, which was 1 × 10^3^ copies/mL. All positive isolates were confirmed by 16S rRNA-PCR and sequenced [6].

### *A. phagocytophilum* PCR

Bacterial DNA was extracted as described above and analyzed by using two different TaqMan real-time PCR assays: the first targeted the *msp2* gene of *A. phagocytophilum* and was adapted from a duplex to a simplex assay [13]. The forward primer (5′-TTGGTCTTGAAGCGCTCGTA) and reverse primer (5′-AATACCATAACCAACACTGCCTTCCAT) generated a 77-bp fragment, which was detected with a TaqMan probe labeled with FAM (5′-CAATCTCAAGCTCAACCCTGGCACCA-MGB). The second PCR targeted the *groEL* gene of *A. phagocytophilum* [14]. The probe was modified to a TaqMan probe (5′FAM-TAACACACTGTGCAATCTTACT-MGB) and the PCR reaction generated a 61-bp-long fragment. Both PCR-reactions contained 1× FastStart Taqman Probe Master (Roche, Basel, Switzerland), 600 nM of each primer, 150 nM of probe, and 5 μL of DNA template. Real-time PCR was performed by using Rotorgene 6000 (QIAGEN, Hilden, Germany). Reaction conditions were 95 °C for 10 min, followed by 45 cycles at 95 °C for 15 s, and 56 °C (53 °C for *groEL*) for 1 min. The limit of detection for both assays was 1 × 10^4^ copies/mL and was established using a synthetic plasmid (Genscript).

## Results

### Participants and study material

There were 18 neoehrlichiosis patients included in the study, all of whom were diagnosed by PCR analysis of blood samples (Table 1). The majority of the patients (17/18) was immunocompromised and had typical risk factors for severe neoehrlichiosis, e.g. an underlying hematologic or systemic autoimmune disease, splenectomy, rituximab treatment, chemotherapy, and/or systemic corticosteroids. Every one of the immunocompromised patients presented with febrile disease, but for one who suffered from nightly sweats and headache (SE16) (Table 1). All of the patients responded completely to treatment with doxycycline and cleared the infection with one exception (SE18), who was given 100 mg doxycycline for 10 days as empiric treatment for unexplained fever instead of the recommended dose of 200 mg doxycycline for 2–3 weeks [1]; the patient in question had PCR-positive and symptomatic recurrence of the infection about 40 days later.

In addition, clinical serum samples originating from 101 anonymous patients submitted to the three clinical laboratories that currently perform *A. phagocytophilum* serology in Sweden and Denmark were analyzed. Whereas the serum samples from County Hospital Ryhov (*n* = 22) and Statens Serum Institut (*n* = 11) were selected based on being positive for *Anaplasma* antibodies, those from Sahlgrenska University Hospital were mostly consecutive samples (*n* = 68) submitted to the laboratory for analysis of *Anaplasma* serology, of which 21 (31%) turned out to have an IgG titer of 1:64 or higher. The serum samples from Sahlgrenska University Hospital were obtained from patients aged between 19 and 80 with a median age of 58, with an even sex distribution (53% women), similar to the serum samples from County Hospital Ryhov (Jönköping), which were from patients 32 to 76 years old (median age 56 years; 45% women). The majority of the serum samples from Statens Serum Institut (Copenhagen) was from women (73%) having an age range of 27 to 67 years and a median age of 53 years.

### *A. phagocytophilum* seropositivity among neoehrlichiosis patients

First, we investigated the blood samples from the 18 patients diagnosed with neoehrlichiosis regarding serologic reactivity to *A. phagocytophilum*. Four of the 18 patients (22%) were *Anaplasma* seropositive at the time of diagnosis (Table 2): the only immunocompetent patient included in the study and 3 out of the 17 who were immunocompromised. One patient had both IgM and IgG antibodies to *Anaplasma* and three had only IgG antibodies. All of the IgG titers were below 1:256.

**Table 2:** Serum antibody titers to *A. phagocytophilum* in neoehrlichiosis patients

Patient ID	IgM	IgG
SE01	1:80	1:160
SE02	N	N
SE03	N	N
SE05	N	N
SE06	N	N
SE09	N	N
SE10	N	1:320
SE12	N	N
SE13	N	N
SE15	N	N
SE16	N	1:80
SE17	N	N
SE18	N	N
SE19	N	N
SE20	N	N
SE21	N	1:160
SE22	N	N
NO01	N	N

*N* negative

### *Ca.* N. mikurensis and *A. phagocytophilum* DNA in the blood of patients with suspected anaplasmosis

Two out of the 101 serum samples queried for *Anaplasma* antibodies were positive for *Ca.* N. mikurensis DNA by PCR (SE15 and SE18; Table 2). Both of these patients had typical symptoms of neoehrlichiosis and a characteristic risk profile (an underlying systemic autoimmune or hematologic disease, rituximab therapy, splenectomy) (Table 1). Neither of these two patients developed antibodies to *A. phagocytophilum* (Table 1). Moreover, not a single one of the 54 patients seropositive for *A. phagocytophilum* was positive in either of the two *A. phagocytophilum* PCRs (data not shown).

### Kinetics of antibody levels to *A. phagocytophilum* in neoehrlichiosis patients

**Fig. 1:**
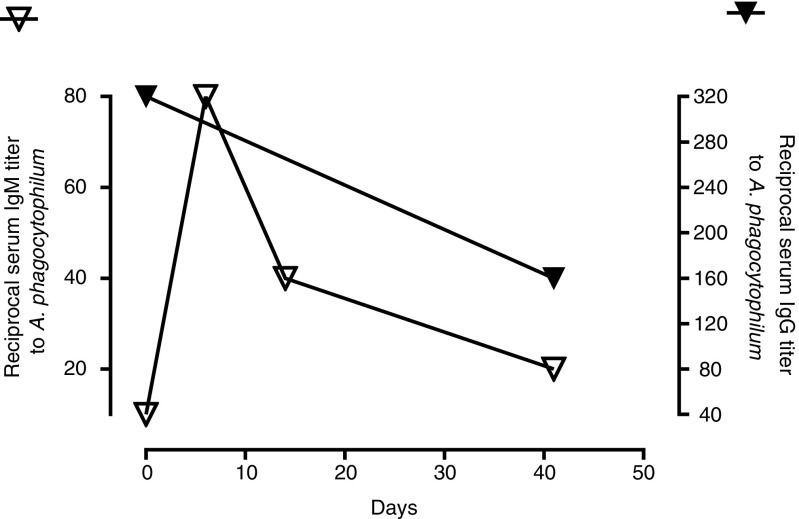
Consecutive *A. phagocytophilum* antibody titers displayed by two neoehrlichiosis patients. One patient (NO01) presented with IgM antibodies (white triangles) and another (SE10) with IgG antibodies (black triangles). Day 0 indicates the day of diagnosis of the *Ca.* N. mikurensis infection

Repeated (paired) serum samples were only available from five of the neoehrlichiosis patients. Three of these patients (SE02, SE13, SE18) were seronegative for *A. phagocytophilum* on both sampling occasions. One patient developed IgM antibodies 1 week after diagnosis (NO01) that decreased 4-fold over a 5-week period (Fig. 1). Another patient (SE10) had IgG antibodies with a titer of 1:320 that was halved over a period of 41 days (Fig. 1).

## Discussion

The main finding of this study is that every fifth of the neoehrlichiosis patients had low titers of *A. phagocytophilum* antibodies in the blood at the time of diagnosis. This is a surprisingly high figure in view of the fact that the majority of the patients were immunocompromised. This seroreactivity to *A. phagocytophilum* might represent (1) previous exposure to or infection with *A. phagocytophilum*, (2) co-infection with *Ca.* N. mikurensis and *A. phagocytophilum*, or (3) *Anaplasma*-crossreactive antibodies elicited or boosted by *Ca.* N. mikurensis infection.

Estimates of the seroprevalence of *A. phagocytophilum* antibodies in the general population in Scandinavia vary greatly. Two older studies report seroprevalence figures of 2.0 and 2.5% among Danish and Norwegian blood donors [15, 16], but a newer Norwegian study gives a figure of 16% [17]. The estimates of the *A. phagocytophilum* seroprevalence in populations that are heavily tick-exposed range from 10% in Norway [15], 11–17% in Sweden [18, 19], to 21% in Denmark [16]. It should be noted that the more recent seroepidemiologic surveys have utilized the same IFA as in the present study, which is based on a human isolate of *A. phagocytophilum* [16, 17, 19], whereas the older studies have used an equine *A. phagocytophilum* isolate [15, 18]. In contrast to the relatively high seroprevalence of *A. phagocytophilum* antibodies, there is a scarcity of case reports of anaplasmosis from the Scandinavian countries [20, 21]. The main explanation for this disparity is that the European variant of this infectious disease is relatively mild in humans, at least compared to human anaplasmosis in North America [22]. Presumably, the human-tropic European *A. phagocytophilum* strains are less virulent than the American ones and give rise to discrete symptoms or only subclinical infections in the majority of cases. Thus, symptomatic anaplasmosis appears to be a rare disease in Scandinavia. This may account for our inability to detect *A. phagocytophilum* DNA in the serum samples derived from the *Anaplasma*-reactive anonymous patient samples, even when using two different PCRs targeting different *A. phagocytophilum* genes. However, we cannot exclude that our use of serum or plasma may have given a poorer DNA yield compared to if we had used buffy coat or whole blood in view of the intracellular nature of *A. phagocytophilum*, which resides within granulocytes.

It is possible that the *Anaplasma* seroreactivity we have detected among neoehrlichiosis patients reflects previous exposure to *A. phagocytophilum.* We have no evidence to suggest that the neoehrlichiosis patients were doubly infected with *A. phagocytophilum* and *Ca.* N. mikurensis since all patients were negative for *A. phagocytophilum* by PCR and confirmatory sequencing of the *Ca.* N. mikurensis PCR amplicons was in no case ambiguous.

The third possible explanation for why up to every fifth of the neoehrlichiosis patients presented with *Anaplasma* antibodies is that these antibodies were in fact directed against *Ca*. N. mikurensis and cross-reactive with *A. phagocytophilum* antigens. One indication that this might be the case is the semblance of an antibody response among the few neoehrlichiosis patients from whom it was possible to obtain repeated blood samples in the present study. Two published studies have also implied that neoehrlichiosis patients may respond with *Anaplasma*-reactive antibodies, indicative of cross-reactivity [2, 6]. However, the issue of cross-reactivity will only be addressable once *Ca.* N. mikurensis antigens are available, which will require its cultivation.

Irrespective of the underlying mechanisms behind the *A. phagocytophilum* seroreactivity demonstrated by some neoehrlichiosis patients, the main significance of this finding is that a certain degree of vigilance is warranted: patients believed to have anaplasmosis may in fact have neoehrlichiosis. Moreover, patients queried for *A. phagocytophilum* antibodies that turn out to be seronegative may have neoehrlichiosis. Two of the neoehrlichiosis patients described in this study were discovered among patient samples submitted for *A. phagocytophilum* serology thanks to relevant clinical data, both of whom were seronegative for *Anaplasma*. A correct diagnosis is of utmost importance since these two infectious diseases differ with regard to one vital aspect: neoehrlichiosis patients have a substantial risk of contracting vascular events such as deep vein thrombosis, arterial aneurysms or transitory ischemic attacks, which are not recognized to be part of an infectious process [5]. If neoehrlichiosis patients with vascular complications are correctly diagnosed and adequately treated with antibiotics, they do not incur new vascular events [5].

To conclude, patients with fever of uncertain origin or with suspected anaplasmosis may in fact have neoehrlichiosis. Misdiagnosed or undiagnosed cases of neoehrlichiosis may be identified either among *Anaplasma* seropositive patients or among patient samples queried for *Anaplasma* antibodies that are negative by *A. phagocytophilum* IFA but have typical risk factors for severe neoehrlichiosis. We recommend that such patients be assayed for the presence of *Ca.* N. mikurensis DNA by PCR performed on EDTA blood or plasma to determine if they have contracted neoehrlichiosis.
